# CMTM6 promotes migration, invasion, and EMT by interacting with and stabilizing vimentin in hepatocellular carcinoma cells

**DOI:** 10.1186/s12967-021-02787-5

**Published:** 2021-03-23

**Authors:** Xiaoting Huang, Leyang Xiang, Baiyao Wang, Jijie Hu, Chunshan Liu, Anbang Ren, Kunpeng Du, Gengtai Ye, Yingying Liang, Yunqiang Tang, Dinghua Yang, Yawei Yuan

**Affiliations:** 1 Department of Radiation Oncology, Affiliated Cancer Hospital & Institute of Guangzhou Medical University, No. 78 Hengzhigang Road, Guangzhou, 510095 Guangdong People’s Republic of China; 2 State Key Laboratory of Respiratory Diseases, Guangzhou Institute of Respiratory Disease, Affiliated Cancer Hospital & Institute of Guangzhou Medical University, Guangzhou, China; 3 Department of Surgery, Affiliated Cancer Hospital & Institute of Guangzhou Medical University, No. 78 Hengzhigang Road, Guangzhou, 510095 Guangdong People’s Republic of China; 4 Unit of Hepatobiliary Surgery, Department of General Surgery, Nanfang Hospital, Southern Medical University, 1838 North Guangzhou Avenue, Baiyun District, Guangzhou, 510515 China; 5 Department of Orthopaedics and Traumatology, Nanfang Hospital, Southern Medical University, Guangzhou, China

**Keywords:** HCC, CMTM6, EMT, Proliferation, Migration, Invasion

## Abstract

**Background:**

CKLF like MARVEL transmembrane domain containing 6 (CMTM6) has been associated with the development in many kinds of cancers. However, the roles of CMTM6 in hepatocellular carcinoma (HCC) are largely unknown. Thus, the present study aimed to investigate the function of CMTM6 in HCC.

**Methods:**

We analysed CMTM6 levels and functions using human HCC cell lines, paired HCC and adjacent non-tumorous tissues, and a tissue microarray. CMTM6 expression was silenced using short hairpin RNAs and its was overexpressed from a lentivirus vector. CMTM6 mRNA and protein levels were determined using quantitative real-time reverse transcription PCR and western blotting, respectively. Proliferation, colony formation, migration, and invasion were assessed using a Cell counting kit-8, colony formation, wound-healing, and Matrigel invasion assays, respectively. Immunohistochemistry was used to score the expression of CMTM6 in tissue samples. The localization and binding partners of CMTM6 were investigated using immunofluorescence and coimmunoprecipitation experiments, respectively. A mouse xenograft model was used for in vivo studies.

**Results:**

Compared with that in adjacent, non-cancerous tissue, Here, CMTM6 levels were increased in HCC tissue samples. Silencing of *CMTM6* suppressed the proliferation, migration, and invasion of HCC cells. Conversely, *CMTM6* overexpression enhanced HCC cell invasion, migration, and proliferation. Mechanistically, CMTM6 physically interacts with and stabilizes vimentin, thus inducing epithelial–mesenchymal transition (EMT), which promotes proliferation, migration and invasion. Importantly, in HCC tissues, CMTM6 expression correlated positively with vimentin levels. Poor prognosis of HCC was associated significantly with higher CMTM6 expression.

**Conclusions:**

CMTM6 has an important function in HCC proliferation, migration, and invasion, via its interaction with and stabilization of vimentin. CMTM6 might represent a potential biomarker and therapeutic target to treat HCC.

**Supplementary Information:**

The online version contains supplementary material available at 10.1186/s12967-021-02787-5.

## Background

Hepatocellular carcinoma (HCC), a major type of liver cancer, is one of the most common malignancies worldwide and is associated with high mortality [1]. HCC is also one of the most common cancers in China, in which 230,000 patients with HCC die each year [2]. Although there have been continuous improvements in medical technology for HCC treatment, the 5-year survival rate of HCC is < 30%, primarily because of early metastasis, representing the major obstacle for HCC treatment [3, 4]. Therefore, is critical to identify the molecular mechanisms involved HCC metastasis.

CKLF like MARVEL transmembrane domain containing 6 (CMTM6) is expressed at the plasma membrane of various cancer cells [5–7]. Recent studies indicated that CMTM6 is an important regulator of programmed cell death 1 ligand 1 (PD-L1); CMTM6 interacts with PD-L1 and prevents its lysosomal degradation, thus stabilising PD-L1 in the membrane [8]. Inhibition of CMTM6 led to decreased PD-L1 protein levels and enhanced tumour-specific T-cell activity [9]. Several studies indicated that CMTM6 expression correlated positively with PD-L1 expression and was associated with poor prognosis of various cancers [7]. Moreover, in neck squamous cell carcinoma cells, CMTM6 is involved in the maintenance of cancer stem cell (CSC) phenotypes and the induction of epithelial–mesenchymal transition (EMT) by transforming growth factor beta (TGFβ), associated with enhanced Wnt/b-catenin signalling [6, 10]. These findings suggest CMTM6 as a promising target molecule in cancer treatment. However, in HCC, the function of CMTM6 largely unknown.

EMT is a cellular process by which epithelial cells gain a mesenchymal phenotype through specific changes in gene expression [6]. EMT-induced changes in epithelial plasticity are evidenced by the loss of epithelial markers, such as E-cadherin, and increased expression of mesenchymal proteins, such as N-cadherin and Vimentin, and the EMT transcription factors such as snail family transcriptional repressor 1 (SNAI1) and Twist family BHLH transcription factor 1 (Twist) [11]. During EMT, there is a restructuring of the epithelial cytoskeleton, resulting in loss of connections with the basement membrane and cell polarity, leading to increased cellular metastatic abilities [12]. Emerging evidence indicated that in certain cancer, such as HCC, EMT participates in metastasis and recurrence [13]. Some studies indicated that HCC cells acquire a more invasive and aggressive CSC phenotype through EMT [14]. Moreover, EMT also implicated in chemoresistance of HCC cells and plays a key role in therapeutic responsiveness of HCC [15]. Therefore, to treat HCC, a promising strategy might be inhibiting metastasis and increasing chemosensitivity by regulating EMT.

The present study aimed to assess the role of CMTM6 in HCC. The results demonstrated upregulation of CMTM6 in HCC tissue samples compared with that in adjacent normal tissues. CMTM6 promotes migration and invasion of HCC cells through inducing EMT. Mechanistically, CMTM6 interacts with and stabilises vimentin, thereby promoting EMT and metastasis of HCC cells.

## Methods

### Cell culture

Human HCC cell lines were obtained from the Cell Bank of Type Culture Collection of the Chinese Academy of Sciences (Shanghai, China). Roswell Park Memorial Institute (RPMI)-1640 medium containing 100 mg/ml streptomycin, 100 U/ml penicillin, and 10% foetal bovine serum (FBS) Gibco, Mulgrave, Australia) was used for cell culture. Cell culture was maintained under standard cell culture conditions in humidified 5% CO_2_.

### Patients and specimens

The present study obtained HCC and adjacent non-tumorous tissues from patients with HCC (n = 40) who received radical resection in Affiliated Cancer Hospital & Institute of Guangzhou Medical University (Guangzhou 510515, China). The patients provided signed informed consent for the use of their collected samples, according to the internal review and ethics boards of the Affiliated Cancer Hospital & Institute of Guangzhou Medical University. Histopathology was used to confirm a diagnosis of HCC.

### Lentiviral construction and transduction

Genechem (Shanghai, China) constructed the U6-sh-CMTM6-CMV-GFP lentiviral vector, which was used to silence *CMTM6* expression. The negative control comprised a lentiviral vector containing a non-silencing short hairpin RNA (shRNA). To silence CMTM6, HCC cells were infected with the lentiviral vector encoding the CMTM6-specific shRNA sequences, control cells were infected with the negative control vector. Puromcyin (Sigma Aldrich, St. Louis, MO, USA) was used to select stable clones for 2 weeks. Expression of the CMTM6 protein was detected using western blotting. Additional file 1: Table S1 shows the shRNA sequences.

### Small interfering RNA (siRNA) transfection

RiBoBio (Guangzhou, China) provided the small interfering RNA (siRNA) targeting *VIM* (encoding vimentin). Cells were seeded into six-well culture plates and grown until they reached 50% confluence (usually the second day). Lipofectamine 3000 reagent (Invitrogen, Waltham, MA, USA) was used to perform the transfections, as described in the manufacturer’s instructions. At 48 h after transfection, the cells were subjected to functional assays. Western blotting was used to evaluate the transfection efficiency. Additional file 2: Table S2 shows the siRNA sequences.

### Quantitative real-time reverse transcription PCR (qRT-PCR)

The TRIzol Reagent (Invitrogen) was used to extract total RNA from HCC cells or tissues, following the manufacturer’s guidelines. A SYBR Green PCR kit (Takara Biotechnology, Dalian, China) was used to perform quantitative real-time PCR (qRT-PCR). Data were analysed and normalized to 18S rRNA expression. Additional file 3: Table S3 shows the primer sequences.

### Western blotting analysis

Cell Signaling Technology (Danvers, MA, USA) provided the primary antibodies recognising glyceraldehyde-3-phosphate dehydrogenase [GAPDH (control), CMTM6, N-cadherin, vimentin and E-cadherin]. Total protein was extracted from HCC tissues or cells by lysis using Radioimmunoprecipitation assay (RIPA) buffer containing proteinase and phosphatase inhibitor cocktails (Sigma Aldrich). The proteins were separated using 8% to 15% SDS-PAGE and then transferred electrophoretically onto a polyvinylidene fluoride membrane (Invitrogen, Carlsbad, CA, USA). The membranes were blocked using 5% bovine serum albumin, washed, incubated with primary antibodies at 4 °C overnight, washed, and incubated with horseradish peroxidase-conjugated goat anti-rabbit or anti-mouse IgG antibody as appropriate. The immunoreactive protein bands were visualised using a chemiluminescence system (Pierce Biotechnology, Rockford, IL, USA).

### Tissue multiarray analysis

Shanghai Outdo Biotech (Shanghai, China) produced tissue microarray sections comprising paired HCC and adjacent non-tumorous tissue samples from 90 patients with HCC. The detailed clinical follow-up data related to outcomes were provided by National Engineering Center Biochip at Shanghai. The follow up began at the date of surgery. Survival was calculated as the time from the baseline date to the clinical outcome diagnosis date or the date of last available clinical record. The patient’s history, physical examination, and laboratory tests were evaluated during follow-up, as needed. The time from surgery to death was defined as overall survival (OS).

### Cell counting kit-8 (CCK-8) assay

The CCK-8 assay was used to assess cell proliferation ability. Cells transfected with siRNAs targeting *CMTM6* were seeded into 96-well plate at of 5 × 10^3^ cells/well. The CCK-8 solution was used to treat the cells at 0, 24, 48, 72, and 96 h. Absorbance at 570 nm was tested using a microplate spectrophotometer from triplicate groups.

### Colony formation assay

Cell seeded into 6-well plates were allowed to grow for 12 days. 4% paraformaldehyde was then used to fix the colonies before staining for 30 min with 0.1% crystal violet. The plates were washed with phosphate-buffered saline (PBS), imaged, and the colonies were counted by visual inspection.

### Wound-healing assay

Equal amounts of cells were plated and allowed to grow to 90% confluence. A sterile pipette tip was used to make a wound in the cell monolayer. The cells on the plate were rinsed gently using medium, which was then replaced with fresh medium. The wound areas were marked and photographed at 0, 12, 24, and 48 h under a phase-contrast microscope. Wound healing was determined by comparison with the wound area at 0 h.

### Cell invasion assay

The cell-invasive assay was performed using 24-well Matrigel invasion chambers with 8 µm-diameter pore inserts (BD Biosciences, Madrid, Spain). Cells grown in serum-free medium (1.5 × 10^5^ cells/100 μl) were added to the top chamber. 10% FBS was added to the bottom chamber as a chemoattractant. Over 24 h, the cells were allowed to migrate through the porous membrane. Then, methanol was used to fix the lower chambers, followed by staining with 0.3% crystal violet for 5 min. After washing with PBS, images were captured under an inverted microscope (Olympus Microscopes, Tokyo, Japan). Cells were counted in five random high power fields in three independent inserts and then averaged.

### Immunofluorescence (IF)

Cells were seeded into glass coverslips in a 12-well plate at a density of 3 × 10^4^ per well. The cells were fixed with paraffin and permeabilized using 0.3% Triton X-100. Subsequently, the fixed cells were incubated with primary antibodies against CMTM6 (Abcam, Cambridge, MA, USA) and Vimentin (Proteintech, Rosemont, IL, USA) overnight at 4 °C, and then incubated with secondary antibodies (Alexa 488 goat anti-Rabbit antibodies; Molecular Probes, Eugene, OR, USA). After counterstaining with Hoechst 33258 (Sigma), images were obtained under a confocal microscopy (Leica Microsystems, Milton Keynes, UK).

### Coimmunoprecipitation (Co-IP)

Myc-tagged vimentin and Flag-tagged CMTM6 expression plasmids were transfected into HCC cells. To precipitate the target proteins, cells were lysed with lysis buffer and incubated with 5 µg of the primary antibody, followed by incubation with a slurry of precleared protein agarose bead (Roche, Mannheim, Germany) at 4 °C for 2 h. After thorough washing, western blotting of the precipitates was used to analyse the potential interacting proteins.

### Xenograft studies

The animal experiments were performed strictly according to the principles and procedures approved by the Committee on the Ethics of Animal Experiments of Guangzhou Medical University (Guangzhou, China). To assess in vivo tumorigenesis, 5 × 10^6^ CMTM6-silenced MHCC-97H cells in of serum-free RPMI 1640 (0.1 ml) were injected into the left shoulder of 4-week-old female BALB/cnu/nu nude mice subcutaneously (four mice in each group). The size of the resulting tumour was measured weekly. The tumour volumes were calculated using the following formula: (length × width^2^)/2.

The mice were sacrificed at 32 days after inoculation, and the tumours were dissected and weighed.

To evaluate the mouse liver metastasis potential of cancer cells, 5 × 10^6^/0.2 ml CMTM6-silenced Bel-74029 cells were injected into BALB/c nude mice tail veins (four mice in each group). After 8 weeks, the mice were sacrificed, and the lung metastatic tumours were dissected for further assays. In another group of mice, at 8 weeks after HCC cell injection, the mice were sacrificed and their lungs were removed. An In-Vivo F Imaging System (Kodak, Rochester, NY, USA) was used to assess the metastatic tissues in the lungs after hematoxylin and eosin (H&E) staining.

### Immunohistochemistry (IHC)

Tissues were fixed in paraformaldehyde and embedded in paraffin. The sections were deparaffinized and hydrated, and then sodium citrate buffer was used to pretreat the sections by heating in a microwave to retrieve the antigen, followed by blocking with normal goat serum. Rabbit anti-CMTM6, anti-Vimentin or anti-Ki67 antibodies (Cell Signaling Technology) were then incubated with the sections at 4 °C overnight. The next day, biotinylated goat anti-rabbit IgG secondary antibodies were incubated with the sections at room temperature (19–21 °C) for 1 h. Lastly, avidin–biotin peroxidase complex (GeneTex, Irvine, CA, USA) was used to stain the sections. Staining intensity was assessed using a semi-quantitative approach (0, negative; 1, weak; and 2, strong) and the percentage of positively stained malignant cells was scored as: 0, 0–4%; 1, 5–24%; 2, 25–49%; 3, 50–74%; and 4, 75–100%. The final IHC scores were determined by multiplying the intensity score by the percentage counts. Positive expression of CMTM6 was defined as having an IHC score ≥ 3.

### Statistical analysis

All statistical data are shown as the mean ± the standard deviation (SD). Student’s t-test or analysis of variance (ANOVA) were used to perform the statistical analyses. To analyse the relation of CMTM6 expression with the clinicopathological characteristics of the patients, the chi-squared test was used. Survival analyses were assessed using the Kaplan–Meier plotter. SPSS 19.0 software (IBM Corp, Armonk, NY, USA) was used for all statistical analyses. P < 0.05 was considered to indicate statistical significance.

## Results

### CMTM6 is upregulate in HCC

**Fig. 1:**
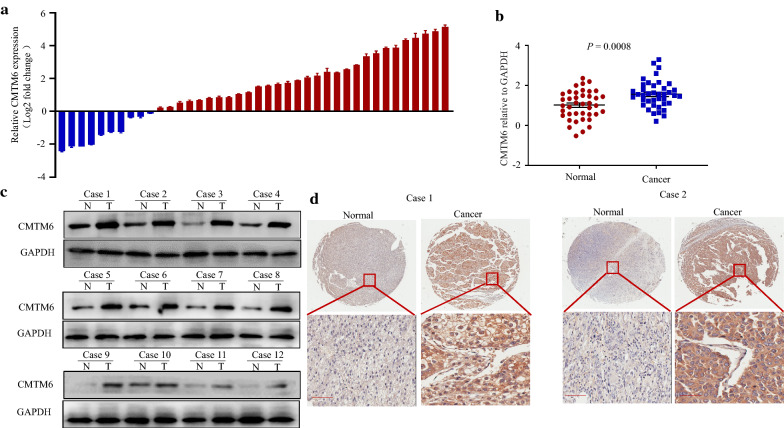
CMTM6 is upregulated in HCC tissues. **a** *CMTM6* expression as assessed using qRT-PCR in 40 pairs of human HCC tissues and adjacent non-cancerous tissues. **b** *CMTM6* expression in HCC tissues was significantly higher than that in the adjacent non-cancerous tissues. **c** Western blotting analysis of CMTM6 protein levels in 40 pairs HCC tissues and adjacent non-cancerous tissues. **d** Immunohistochemical staining of CMTM6 in representative HCC tissue and adjacent non-cancerous tissues. Scale bars, 25 μm

First, we used qRT-PCR to detect the mRNA expression of *CMTM6* in 40 paired human HCC tissues and matched adjacent normal tissues, which showed that the relative mRNA expression *CMTM6* was upregulated in 75% (30/40) of patients with HCC (Fig. 1a). *CMTM6* mRNA expression was increased significantly in HCC tissues compared with that in the adjacent normal tissues (Fig. 1b). Furthermore, CMTM6 levels were upregulated significantly in 12 pairs HCC tissues compared with the adjacent normal tissues, as assessed using western blotting (Fig. 1c). Moreover, IHC results also revealed that CMTM6 expression was higher in HCC tissues compared with that in the matched adjacent normal tissues (Fig. 1d). These results indicated upregulated expression of CMTM6 in HCC tissues.

### CMTM6 promotes HCC cell proliferation, migration, and invasion in vitro

**Fig. 2:**
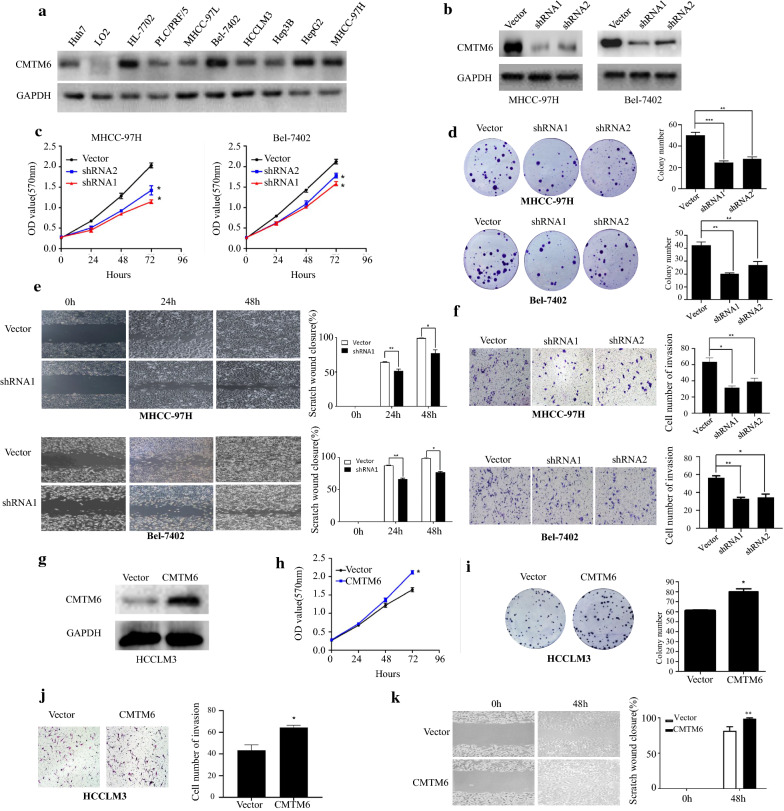
CMTM6 promotes proliferation, migration, and invasion of HCC cells. **a** Western blotting analysis of CMTM6 levels in HCC cell lines. **b** Western blotting analysis of CMTM6 levels in MHCC-97H and BEL-7402 cells. MHCC-97H and BEL-7402 cells were transduced with a lentivirus encoding a *CMTM6* shRNA or scrambled shRNA. **c** Cell proliferation was determined using the CCK-8 assay in HCC cells expressing the *CMTM6* shRNA. **d** Colony-forming efficiency of HCC cells. The cells were transduced with lentivirus encoding the *CMTM6* shRNA or scrambled shRNA. **e** The cellular migration abilities were examined using a wound healing assay in HCC cells expressing the *CMTM6* shRNA. **f** Cell invasion was evaluated using Transwell assays in in HCC cells expressing the *CMTM6* shRNA. **g** Western blotting analysis of CMTM6 expression in HCCLM3 cells transduced with lentivirus expressing CMTM6 or with the empty vector. **h** Cell proliferation was determined using the CCK-8 assay in HCCLM3 overexpressing *CMTM6*. **i** Colony-forming efficiency of HCCLM3 cells overexpressing *CMTM6*. **j** Cell invasion was examined using Transwell assays in HCCLM3 cells overexpressing CMTM6. **k** Wound healing assay

Given that CMTM6 is upregulated in HCC, to investigate the biological functions of CMTM6 in HCC cells, we performed loss-and gain-of-function experiments. First, we determined that CMTM6 is commonly expressed in HCC cells (Fig. 2a). Next, MHCC-97H and BEL-7402 cells with high expression of CMTM6 were transduced with lentiviruses encoding the *CMTM6* shRNA to inhibit *CMTM6* expression (Fig. 2b). Interestingly, silencing *CMTM6* decreased the proliferative capacity of HCC cells significantly, as assessed using CCK-8 and colony formation assays (Fig. 2c, d). Moreover, *CMTM6* silencing decreased HCC cell invasion and migration, according to Transwell and wound-healing assays (Fig. 2e, f). By contrast, overexpression of *CMTM6* in HCCLM3 cells with low endogenous CMTM6 expression increased the proliferative capacity of HCCLM3 cells significantly (Fig. 2g–i). Importantly, Transwell assays showed that overexpression of *CMTM6* enhanced the invasion of HCCLM3 cells (Fig. 2j). Similarly, in a wound-healing assay, overexpression of *CMTM6* led to increased migration potential (Fig. 2k). Thus, in HCC cells, CMTM6 enhances invasion, migration, and proliferation.

### CMTM6 silencing reduces in vivo HCC cell proliferation and tumour metastasis

**Fig. 3:**
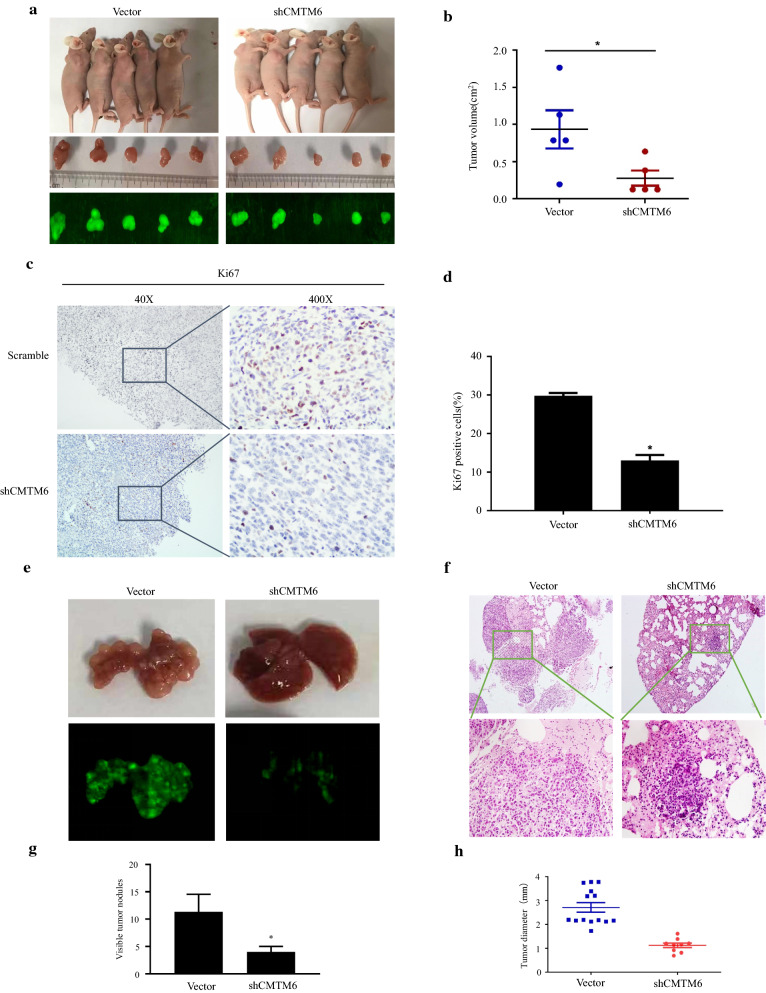
Knockdown of *CMTM6* inhibits HCC cell proliferation and metastasis in vivo. **a** Images of mice injected with *CMTM6*-silenced MHCC-97H cells. **b**, **c** IHC and Ki-67 expression in tumours from mice injected with HCC cells. **d** Tumour weight of model mice; the tumours were lighter in the mice that received cells silenced for *CMTM6* expression than in those that received cells transduced with the empty vector. Tumour weights are indicated by the data points. **e** Fluorescence images of metastatic lung nodules of mice injected with Bel-7402 cells silenced for *CMTM6*. **f** Representative H&E staining of lung metastasis nodules. **g** The number of lung metastatic loci. Student t test was used to analyse the number of metastatic nodules. **h** The expression of Ecadherin in tumors was determined using quantitative PCR (qPCR). **p < 0.001, vector vs. shCMTM6

To investigate the in vivo effect of CMTM6 on tumour growth, lentivirus-mediated shRNA targeting CMTM6-transduced cells and control MHCC-97H cells were injected subcutaneously into nude mice’s right shoulders (Fig. 3a). Four weeks later, the size of the tumour in the *CMTM6* knockdown group was considerably decreased compared with that in the control group (Fig. 3b). We observed that in the *CMTM6* knockdown group, the tumour cells were arranged more loosely (as shown by H&E staining), and the expression of Ki-67 had decreased significantly (Fig. 3c, d). These results indicated that knockdown of CMTM6 inhibits the growth and tumorigenicity of HCC cells in vivo*.*

Next, to determine whether CMTM6 could promote in vivo metastasis, we performed a tail vein metastatic assay in nude mice using Bel-7402 cells that were transduced with lentivirus encoding the *CMTM6* shRNA or empty vector. Five-week-old nude mice were injected via their tail-veins with Bel-7402 cells with *CMTM6* knockdown or control cells. At 6 weeks post-injection, the *CMTM6* silenced group had fewer metastatic lesions in their lungs compared with those in the mice who received the control cells (Fig. 3e). Finally, The lung metastatic nodules of the control group mice became larger and larger, while the mice injected with shCMTM6 cells had fewer and fewer metastatic nodules (Fig. 3f, g). These results indicated that knockdown of *CMTM6* reduces the metastatic potential of HCC cells in vivo.

### CMTM6 induces EMT in HCC cell

**Fig. 4:**
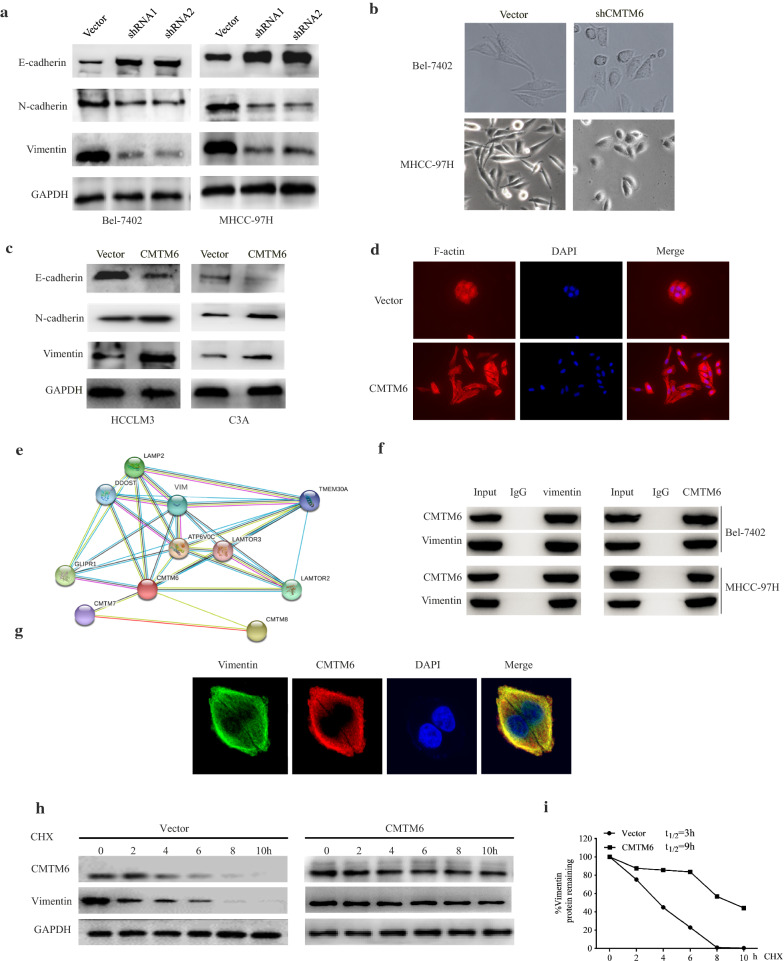
CMTM6 promotes EMT in HCC cells. **a** The levels of vimentin, N-cadherin, and E-cadherin in HCC cells silenced for *CMTM6* expression, as assessed using western blotting. **b** Representative morphology of MHCC-97H cells silenced for CMTM6 expression. **c** The levels of vimentin, N-cadherin, and E-cadherin in HCCLM3 and C3A cells overexpressing *CMTM6*, as assessed using western blotting. **d** F-actin filament staining using rhodamine phallotoxin staining for 48 h in HCCLM3 cells stably overexpressing CMTM6 or empty vector, as assessed using fluorescence microscopy. **e** STRING database analysis to predict potential binding partners of CMTM6. **f** The interaction between CMTM6 and vimentin in Bel-7402 and MHCC-97H cells, as determined using Co-IP. Anti-CMTM6 or anti-Vimentin antibodies were used to immunoprecipitate the lysates. Western blotting was used to analyse the input and the immunoprecipitates using the indicated antibodies. **g** CMTM6 and vimentin double staining HCC cells, as assessed using confocal microscopy. **h**, **i** CHX (20 μg/ml) treatment for various times of HCCLM3 cells overexpressing CMTM6 or transduced with the empty vector. Western blotting was used to analyse the proteins in whole-cell extracts

Tumour invasion and metastasis depend on EMT; therefore, we detected the levels of EMT markers to assess the effect of *CMTM6* knockdown on EMT. *CMTM6* knockdown enhanced the levels of the epithelial marker E-cadherin, but decreased the levels of the mesenchymal markers vimentin and N-cadherin (Fig. 4a). Moreover, *CMTM6* knockdown resulted in morphological change to HCC cells, which underwent a transformation from a mesenchymal phenotype to an epithelial phenotype (Fig. 4b). Conversely, *CMTM6* overexpression decreased E-cadherin levels, and increased vimentin and N-cadherin levels in HCC cells (Fig. 4c). Consistently, cells overexpressing *CMTM6* exhibited a spindle shape and stretched F-actin fibres, which are characteristic of the EMT process (Fig. 4d). These results indicated that CMTM6 promotes EMT in HCC cells.

### CMTM6 interacts with vimentin to induce EMT and promote metastasis of HCC cells

To investigate how CMTM6 induces EMT in HCC cells, the STRING database was used to perform protein–protein interaction analysis. Interestingly, this analysis identified that vimentin might interact with CMTM6 (Fig. 4e). Vimentin, expressed by interstitial cells, decreases adhesion between tumour cells, and increases their invasiveness and migration [16]. Upregulation of vimentin induces EMT, thus contributing to migration and invasion [17]. For instance, FOXK1 interacts with and stabilises vimentin, thereby promoting gastric cancer cell migration and metastasis via the induction of EMT [18]. We hypothesized that CMTM6 interacts with and stabilises vimentin, which in turn induces EMT and promotes the migration and invasion of HCC cells. To verify the physical interaction between CMTM6 and vimentin, reciprocal coimmunoprecipitation experiments were performed. The results confirmed that vimentin and CMTM6 interacted in HCC cell lysates (Fig. 4f). Moreover, vimentin and CMTM6 were observed to colocalise in HCC cells under confocal fluorescence microscopy (Fig. 4g).

To determine whether CMTM6 increases the stability of vimentin, protein synthesis was inhibited in HCC cells using cycloheximide (CHX), and the remaining vimentin level was analysed using western blotting. The level of vimentin in cell overexpressing *CMTM6* remained higher than that in the control cells (Fig. 4h). Importantly, CMTM6 overexpression caused the half-life of endogenous vimentin to increase from 4 to almost 9 h (Fig. 4i). These results suggested that CMTM6 overexpression stabilised vimentin.

**Fig. 5:**
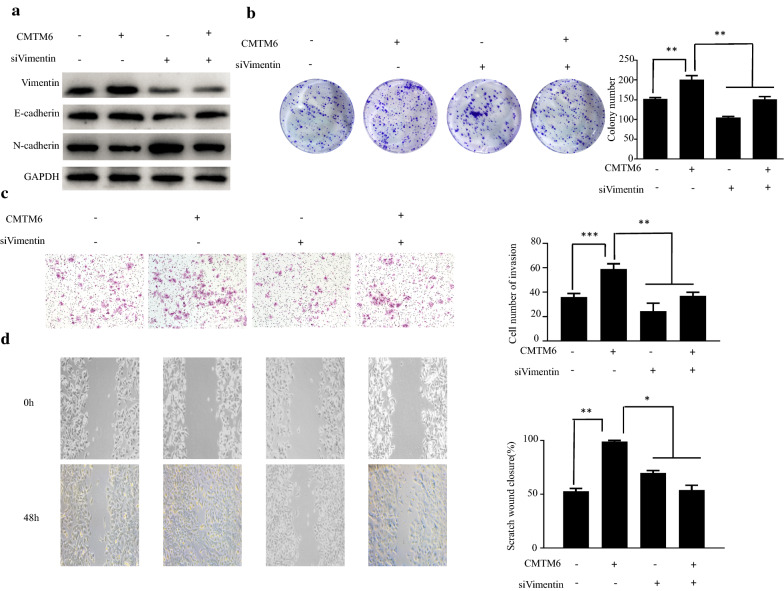
CMTM6 interacts with vimentin to promotes HCC cell EMT. **a** Western blotting analysis of the levels of vimentin, N-cadherin, and E-cadherin in HCCLM3 cells co-transfected with *CMTM6* or *VIM* siRNAs. **b** Colony-forming efficiency of HCCLM3 cells. HCCLM3 cells were co-transfected with *CMTM6* or *VIM* siRNAs. **c** The invasive activity of HCCLM3 cells were evaluated using Transwell assays. HCCLM3 cells were co-transfected with *CMTM6* or *VIM* siRNAs

We sought to confirm whether CMTM6 could regulate EMT through vimentin. After *VIM* was silenced in cells overexpressing *CMTM6*, the expression of EMT epithelial marker, E-cadherin, increased (Fig. 5a), which suggested that suppression of vimentin blocked the EMT induced by *CMTM6* overexpression. Furthermore, a colony formation assay showed that the induction of proliferation by *CMTM6* overexpression was blocked by vimentin inhibition (Fig. 5b). Moreover, Transwell assays showed that knockdown of *VIM* reversed CMTM6-induced invasion and migration (Fig. 5c, d). Collectively, the findings showed that CMTM6 enhances proliferation, metastasis, and EMT at least partly dependent on vimentin.

### Co-expression of CMTM6 and vimentin correlates with poor prognosis in HCC

**Fig. 6:**
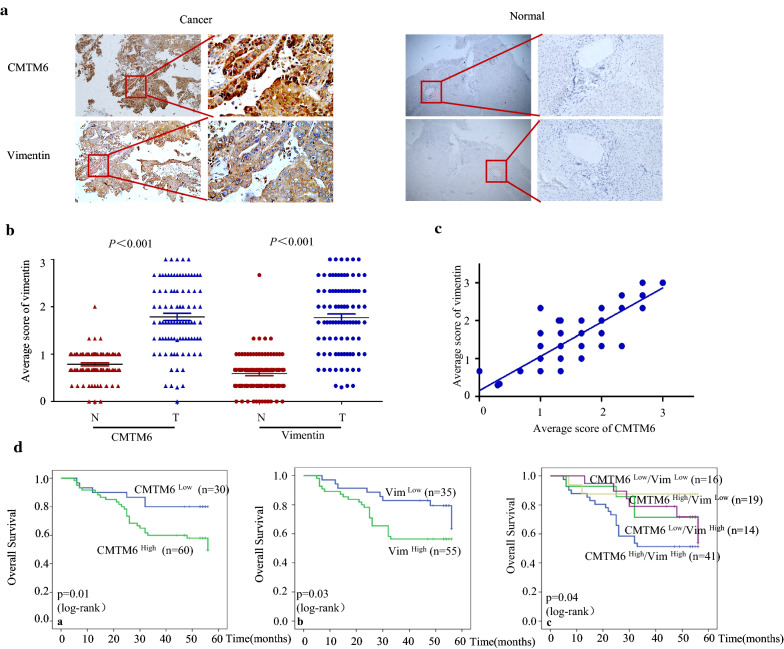
Co-expression of vimentin and CMTM6 correlates with poor prognosis in HCC. **a** IHC analysis of CMTM6 and vimentin levels in in HCC tissues. **b** The average staining scores in cancerous and normal tissues of CMTM6 and vimentin. **c** Quantification of positive staining for CMTM6 and vimentin, and Spearman rank correlation coefficient analysis of their correlation. **d** Overall survival analysis of CMTM6 alone, vimentin alone, combined CMTM6/vimentin expression using the Kaplan–Meier method

We analysed the associated clinicopathological features in HCC to determine the clinical relevance of CMTM6 with vimentin. CMTM6 and vimentin proteins levels were significantly higher in HCC tissues compared with those in adjacent noncancerous tissues (Fig. 6a, b). CMTM6 and vimentin expression correlated positively in HCC specimens (*P* < 0.001) (Fig. 6c). Importantly, CMTM6 was significantly associated with TNM stage (*P* = 0.048), HbsAg (*P* = 0.012), tumour differentiation (*P* = 0.039), tumour size (*P* = 0.024), and microvascular invasion (*P* = 0.04). Vimentin expression correlated markedly with TNM stage (*P* = 0.003), tumour differentiation (*P* = 0.001), tumour size (*P* = 0.024), and microvascular invasion (*P* = 0.013) (Table 1).

**Table 1:** Correlation between CMTM6/vimentin protein expression and the clinicopathological characteristics of patients with HCC (n = 90)

Features	Total number (n = 90)	Expression of CMTM6		*P*-value	Expression of vimentin		*P*-value
		Low	High		Low	High	
Age(years)							
< 60	68	21 (30.9%)	47 (69.1%)	0.386	24 (35.3%)	44 (64.7%)	0.219
≥ 60	22	9 (40.9%)	13 (59.1%)		11 (50.0%)	11 (50.0%)	
Gender							
Male	80	27 (33.8%)	53 (66.3%)	0.813	32 (40.0%)	48 (60.0%)	0.541
Female	10	3 (30.0%)	7 (70.0%)		3 (30.0%)	7 (70.0%)	
Microvascular invasion							
Present	54	23 (42.6%)	31 (57.4%)	0.04*	26 (48.1%)	35 (51.9%)	0.013*
Absent	26	5 (19.2%)	21 (80.8%)		5 (19.2%)	21 (80.8%)	
Tumour size(cm)							
< 5	51	22 (43.1%)	29 (56.9%)	0.024*	25 (49.0%)	26 (51.0%)	0.024*
≥ 5	39	8 (20.5%)	31 (79.5%)		10 (25.6%)	29 (74.4%)	
Differentiation							
Well	15	9 (60.0%)	6 (40.0%)	0.039*	12 (80.0%)	3 (20.0%)	0.001*
Moderate	38	10 (26.3%)	28 (73.7%)		11 (28.9%)	27 (71.1%)	
Poor	31	8 (25.8%)	23 (74.2%)		8 (25.8%)	23 (74.2%)	
HBsAg							
Negative	19	11 (57.9%)	8 (42.1%)	0.012*	6 (31.6%)	13 (68.4%)	0.436
Positive	70	19 (27.1%)	51 (72.9%)		29 (41.4%)	41 (58.6%)	
Serum AFP level (ng/ml)							
< 25	39	9 (23.1%)	30 (76.9%)	0.061	16 (41.0%)	23 (59.0%)	0.772
≥ 25	50	21 (42.0%)	29 (58.0%)		19 (38.0%)	31 (62.0%)	
TNM stage							
I + II	78	29 (37.2%)	49 (62.8%)	0.048*	35 (44.9%)	43 (55.1%)	0.003*
III + IV	12	1 (8.3%)	11 (91.7%)		0 (0.0%)	12 (100.0%)	

* *p* < 0.05

Moreover, univariate Cox regression analysis indicated that microvascular invasion (95% confidence interval (CI) 1.162–5.277; *P* = 0.019), differentiation (95% CI 1.218–3.830; *P* = 0.008), TNM stage (95% CI 1.088–5.407; *P* = 0.030), CMTM6 expression (95% CI 0.997–5.906; *P* = 0.041), and vimentin expression (95% CI 1.007–5.005; *P* = 0.048) could predict OS in patients with HCC. Subsequent multivariate Cox regression analysis showed that differentiation (95% CI 0.309–0.856; *P* = 0.011), CMTM6 (95% CI 1.118–7.096; *P* = 0.006), vimentin (95% CI 1.391–7.340; *P* = 0.037) remained predictive factors. Interestingly, the combined expression of CMTM6 and vimentin (95% CI 0.826–3.908; P = 0.040) was identified as an independent prognostic factor for OS (Table 2) in patients with HCC. The Kaplan–Meier analysis for combined CMTM6 and vimentin revealed that patients with higher CMTM6 and vimentin levels had the shortest post-operative OS (Fig. 6d).

**Table 2:** Univariate and multivariate analyses of different prognostic factors in 90 patients with HCC

Features	Univariate analysis		Multivariate analysis	
	HR (95% CI)	P-value	HR (95% CI)	P-value
Age (years; < 60 vs. ≥ 60)	1.298 (0.600–2.807)	0.508		
Sex (male vs. female)	0.520 (0.124–2.176)	0.371		
Microvascular invasion (absent vs. present)	2.476 (1.162–5.277)	0.019*		
Tumour size (cm; < 5 vs. ≥ 5)	1.928 (0.952–3.904)	0.068		
Differentiation (well, moderate, poor)	2.160 (1.218–3.830)	0.008*	0.514 (0.309–0.856)	0.011*
HBsAg (positive vs. negative)	1.270 (0.516–3.124)	0.603		
AFP (< 25 vs. ≥ 25)	1.607 (0.770–3.356)	0.206		
TNM stage (I + II vs. III + IV)	2.425 (1.088–5.407)	0.030*		
Expression of CMTM6 (low vs. high)	2.427 (0.997–5.906)	0.041*	2.816 (1.118–7.096)	0.006*
Expression of vimentin (low vs. high)	2.245 (1.007–5.005)	0.048*	3.196 (1.391–7.340)	0.037*
Co-expression				
CMTM6 (high) vimentin (high) CMTM6 (high) vimentin (low) CMTM6 (low) vimentin (high) CMTM6 (low) vimentin (low)	0.716 (0.519–0.987)	0.042*	1.796 (0.826–3.908)	0.040*

* *p* < 0.05

## Discussion

The results of the present study revealed upregulation of CMTM6 in HCC tissue samples compared with that in adjacent normal liver tissues. Silencing of *CMTM6* expression suppressed the proliferation, migration, and invasion of HCC cell, whereas CMTM6 had the opposite effects. CMTM6 interacts and stabilises vimentin to induce EMT of HCC cells, thereby promoting proliferation, invasion, and migration. Poorer prognosis if HCC is associated significantly with higher CMTM6 expression. Thus, the CMTM6-vimentin axis has an important function in HCC.

Previous studies showed that CMTM6 participates in tumorigenesis [7, 19]. Herein, IHC and western blotting were used initially to detect CMTM6 expression in HCC samples. CMTM6 was highly expressed in HCC, which suggested that CMTM6 overexpression might be associated with HCC tumour progression. Subsequently, we found that silencing of *CMTM6* markedly inhibited HCC cell invasion and migration and *CMTM6* overexpression had the opposite effects. Importantly, in the xenograft mouse model, *CMTM6* knockdown markedly reduced subcutaneous solid tumour growth and the number of pulmonary metastasis nodules. These data indicated that CMTM6 promotes HCC progression and development. Many studies have explored the relationship between CMTM6 and tumour immunity, indicating that CMTM6 as a promising target for cancer immunotherapy [8, 9]. The results of the present study implicated CMTM6 in the migration and invasion of HCC cells, thus improving our understanding of CMTM6’s function in tumours and suggesting that CMTM6 is a therapeutic target to treat HCC.

Cancer cell migration and invasion rely on EMT, during which epithelial markers (E-cadherin, ZO-1, and Occludin) are downregulated, whereas mesenchymal markers (vimentin, N-cadherin, and Fibronectin) are upregulated [20]. A previous study reported that CMTM6 is involved in EMT regulation in head and neck squamous cell carcinoma [6]; however, whether CMTM6 is implicated in EMT in HCC cells is largely unknown. In this study, we observed that knockdown of *CMTM6* enhanced the expression of E-cadherin, epithelial marker, but decreased the expression vimentin and N-cadherin (mesenchymal markers). By contrast, CMTM6 overexpression decreased E-cadherin expression and increased vimentin and N-cadherin expression. Morphologically, HCC cells silenced for *CMTM6* expression resembled epithelial cells, while the control cells maintained a spindle-like mesenchymal cell morphology. Therefore, these results indicated that CMTM6 promotes EMT in HCC cells.

Vimentin is considered as a protein marker of EMT reprogramming, in which cancer cells acquire migratory and invasive phenotypes [16, 21]. Increased expression of vimentin has been observed in colorectal, breast, gastric, and hepatic cancers [22–25]. High expression of vimentin correlates with the aggressiveness and poor clinical outcome in many types of cancers [26, 27]. Therefore, targeting vimentin is a promising approach for anti-cancer therapy. Several factors were reported to be involved in the regulation of vimentin expression [28–30]. For example, EMT-related transcription factors nuclear factor kappa B (NF-κB) and Jun proto-oncogene, AP-1 transcription factor subunit (AP-1/jun) can bind to the *VIM* promoter and induce vimentin expression, thus promoting tumour cell migration and invasion [30, 31]. Circular RNs circ-10720 increased the vimentin expression levels and promoted EMT by sponging microRNAs that target *VIM* (17). Importantly, circ-10720 expression correlated positively with vimentin expression in patients with HCC [32].

The present study revealed that CMTM6 overexpression enhanced the invasion, proliferation, and EMT of HCC cells via its interaction and stabilisation of vimentin. Our data indicated that CMTM6-induced migration, invasion, and EMT could be blocked by *VIM* knockdown, suggesting that CMTM6 promotes migration, invasion and EMT, at least partly dependent on vimentin. Importantly, we detected a positive correlation between the expression of CMTM6 and vimentin in HCC tissues. The survival time of the patients with positive CMTM6 and vimentin expression was significantly shorter than that of patients with negative expression. Therefore, in HCC, the synergistic effects of vimentin and CMTM6 might be especially important. In addition, the CMTM6 or vimentin level, or both, might be developed as biomarkers for the high likelihood of poor prognosis and tumour metastasis in patients with HCC. Further investigation is required to determine CMTM6′s specific regulatory mechanism in the stabilization of vimentin, the progression of EMT, and the vimentin-interacting region of CMTM6 in HCC cells.

## Conclusions

The results demonstrated that CMTM6 was upregulated in HCC tissue samples compared with that in adjacent normal liver tissues. CMTM6 contributed to migration, invasion, and EMT of HCC cells. Mechanistically, CMTM6 physically interacts with and stabilizes vimentin, thus inducing EMT and promoting proliferation, migration, and invasion. We identified a significant association between poorer prognosis of HCC and higher CMTM6 expression. Our findings indicated that CMTM6 has important functions in the growth and metastasis of HCC through its interaction with, and stabilisation of, vimentin. Thus, CMTM6 could be developed as a biomarker and target molecule for HCC therapy.

## Supplementary Information

**Additional file 1: Table S1.** Sequences of shRNAs.

**Additional file 2: Table S2.** Sequence of siRNAs (5′ to 3′).

**Additional file 3: Table S3.** Sequences of qRT-PCR primers.

## Data Availability

All data generated or analyzed during this study are included in this published article.

## Abbreviations

HCC: Hepatocellular carcinoma
CMTM6: CKLF Like MARVEL Transmembrane Domain Containing 6
qRT-PCR: Quantitative real-time reverse transcription PCR
IHC: Immunohistochemistry
IF: Immunofluorescence
Co-IP: Co-immunoprecipitation
EMT: Epithelial–mesenchymal transition
CCK-8: Cell counting kit-8
siRNA: Small interfering RNA
CSCs: Cancer stem cells
shRNA: Short hairpin RNA
